# Supplementary material for: APC and TP53 Mutations Predict Cetuximab Sensitivity across Consensus Molecular Subtypes
Source: Cancers (Basel). 2021 Oct 27;13(21):5394. doi: 10.3390/cancers13215394 (PMC8582550; doi:10.3390/cancers13215394)
Supplement: Supplementary file 1 [file cancers-13-05394-s001.zip › cancers-1376298-supplementary.pdf]

# Supplementary Figures S1–S4 and Table S1

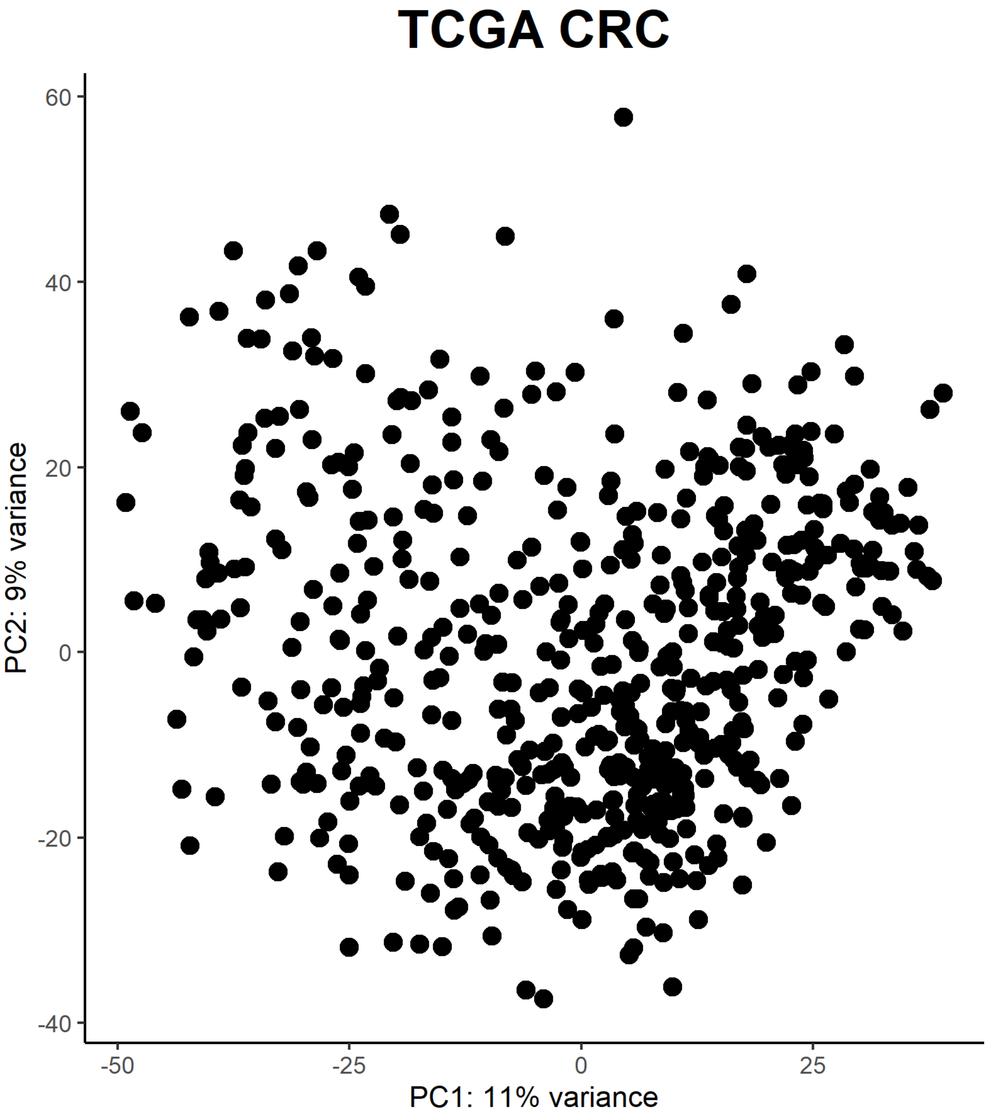


A

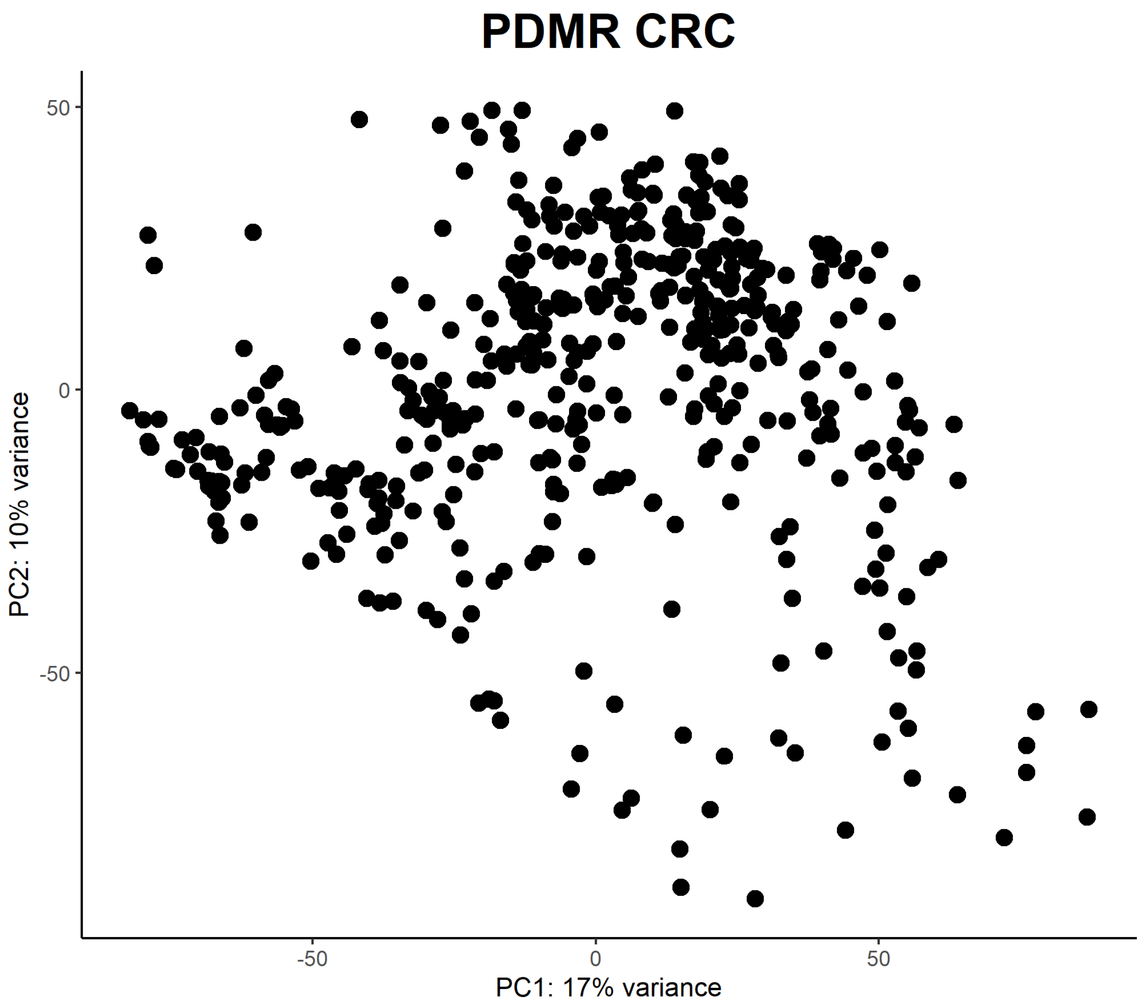


B

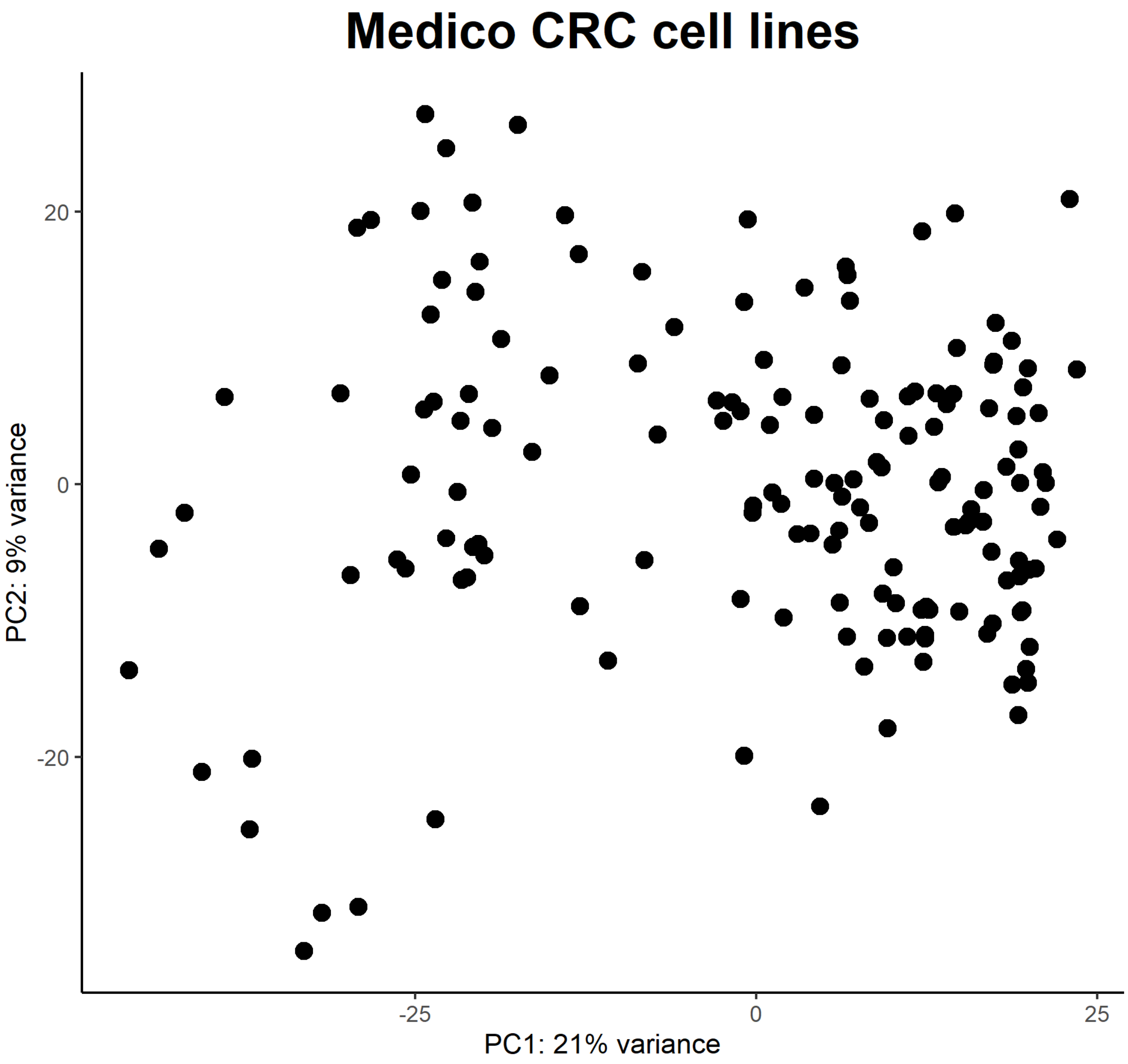


C

**Figure S1.** PCA quality control analysis. PCA plots of gene expression data show the relationship between samples (dots). (**A**) PC2 vs PC1 for TCGA samples (**B**) PC2 vs PC1 for PDMR samples (**C**) PC2 vs PC1 Medico samples. Principle components were generated using the top 1000 variable genes after normalization (RNAseq data is median log2 normalized, array data is Loess log2 normalized). No outliers were detected.

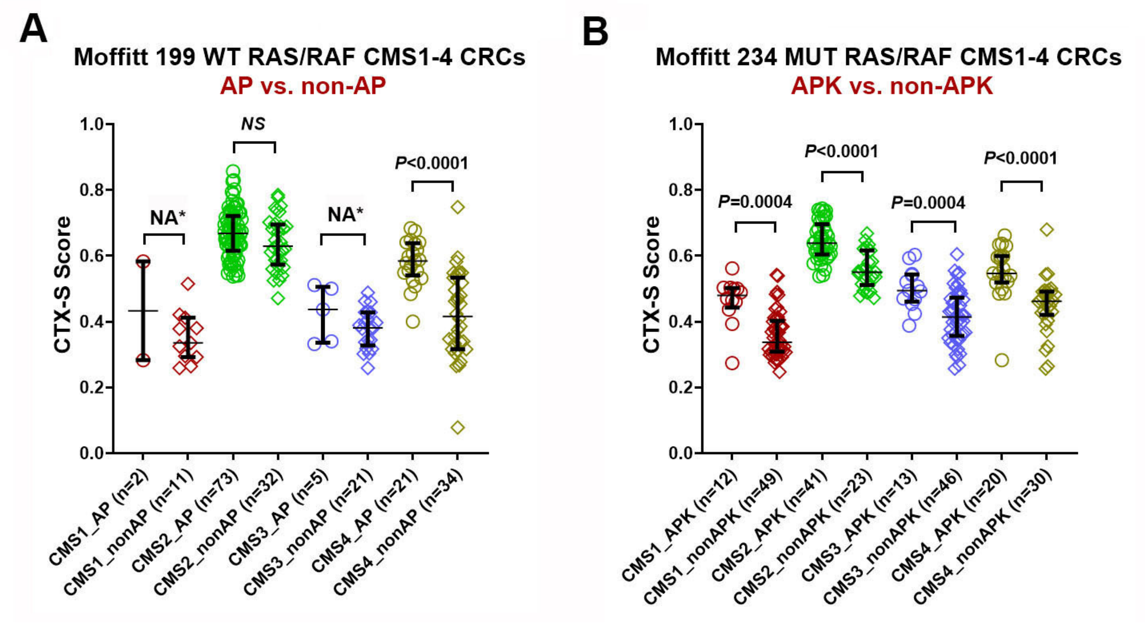


**Figure S2.** Comparison of CTX-S scores in MUT A + P vs nonA + P in each of four CMS classes in (**A**) Moffitt 199 CMS1-4 CRCs with WT RAS/RAF and (**B**) Moffitt 234 CMS1-4 CRCs with MUT RAS/RAF**.** Bars represent Median with interquartile range. P values are for two-tailed Welch t test. NS – not significant; NA* – not applicable for comparison due to small sample sizes of AP tumors (n<10).

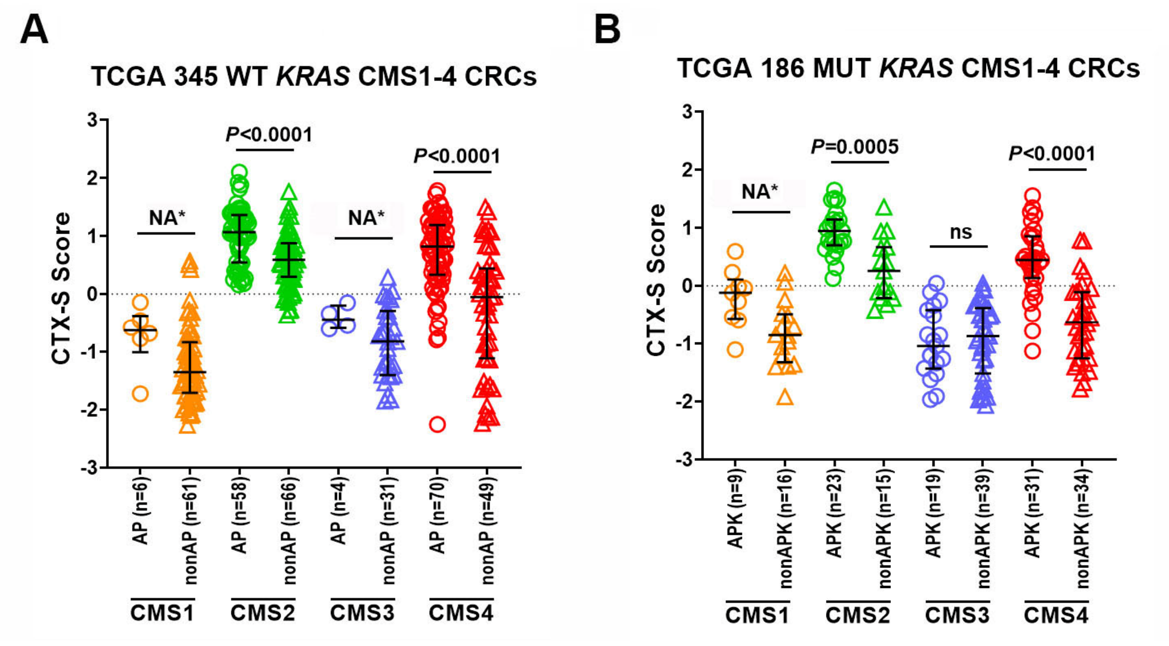


**Figure S3.** (**A**) Comparison of CTX-S scores in AP vs nonAP in each of four CMS classes in TCGA 345 WT *KRAS* CRC tumors. (**B**) Comparison in APK vs nonAPK in TCGA 186 MUT *KRAS* CRC tumors. Bars represent Median with interquartile range. P values are for two-tailed Welch t test. NA* – not applicable for comparison due to small sample sizes of AP tumors (n<10).

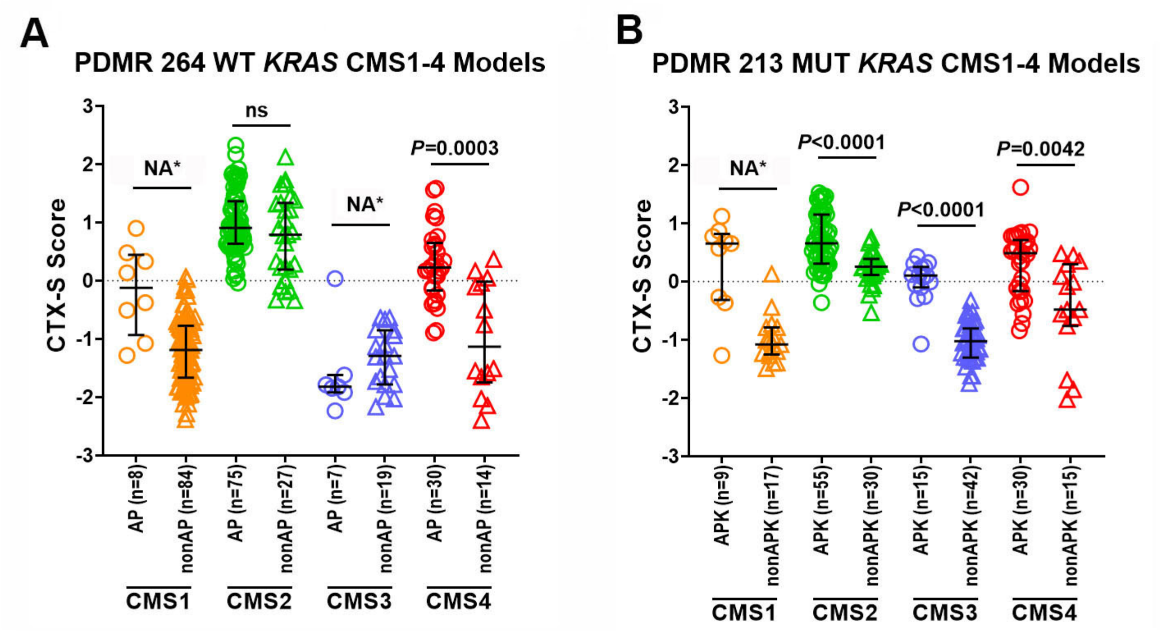


**Figure S4.** (**A**) Comparison of CTX-S scores in AP vs nonAP in each of four CMS classes in PDMR 264 WT *KRAS* CRC models. (**B**) Comparison in APK vs nonAPK in PDMR 213 MUT *KRAS* CRC models. Bars represent Median with interquartile range. P values are for two-tailed Welch t test. NA* – not applicable for comparison due to small sample sizes of AP tumors (n<10).

**Table S1 Pairwise comparison of ages across CMS classes for Moffitt CRCs**

| Pairwise Comparison | lwr95% | upr95% | adj P |
|---|---|---|---|
| CMS2-CMS1 | -13.0394308446566 | -4.09074667013624 | 6.769414798935e-06 |
| CMS3-CMS1 | -10.0975046114328 | 0.10926931731513 | 0.057693938678395 |
| CMS4-CMS1 | -11.9200676292282 | -2.15685544769484 | 0.00129351293568858 |
| CMS3-CMS2 | -0.697273530188256 | 7.8392157508635 | 0.136873847489627 |
| CMS4-CMS2 | -2.47380173664554 | 5.52705617451535 | 0.758518912035925 |
| CMS4-CMS3 | -6.73777048856607 | 2.64908270576064 | 0.675270265155125 |
